# Domestic Correspondence

**Published:** 1873-02-15

**Authors:** William Massie

**Affiliations:** Grandview, Ill.


### Domestic Correspondence.

GRANDVIEW, Ill., Jan. 8th, 1873.

EDITORS MEDICAL EXAMINER:—At a regular meeting of the Esculapian Society of the Wabash Valley, held at Tuscola, Ill., Nov. 27th and 28th, 1872, the following resolutions were unanimously adopted :

*Section* 1st. That no member of this Society shall receive a student in medicine until he shall have been examined by the Board of Censors, and received a certificate of qualification, signed by at least three of their number, and countersigned by the Secretary of this Society.

*Sec.* 2d. That it shall be the duty of the Censors to examine applicants for the study of medicine as to their literary and other qualifications, subject to such rules and regulations as may be prescribed by the Society; and they shall give a certificate of qualification to such as pass a satisfactory examination.

*Sec.* 3d. It shall be the duty of the Secretary to countersign the certificates of qualification of medical students given by the Censors, and enter such certificate into the published minutes of the Society.

*Sec.* 4th. Students must satisfy the Censors, by certificate or diploma from competent literary schools, or by examination, that they possess a good elementary knowledge of the following English branches, viz.: Orthography, Reading, English Grammar and Composition, Arithmetic, Algebra, Geometry, and Plane Trigonometry, Botany, Natural Philosophy, and Inorganic Chemistry; and enough Latin to enable them to read, write, or translate correctly any Latin or English prescription given them.

*Sec.* 5th. The Secretary shall, upon the order of the Society, give students who have received certificates of qualification a certificate setting forth the length of time that they have devoted to the study of medicine, under whom, and that he is a regular practitioner of medicine in good professional standing, which certificate shall also be entered into the published minutes of the Society.

Comment by us is hardly necessary; but we trust that this is an initiatory step in the right place and direction to remove some of the difficulties that complicate the question of medical education. That time and experience may require the above resolutions to be greatly modified, there can be little doubt; but everything must have a beginning.

Any criticisms or suggestions will be thankfully received.

Respectfully yours, etc.,

WILLIAM MASSIE, M.D.

